# Could SARS-CoV-1 Vaccines in the Pipeline Have Contributed to Fighting the COVID-19 Pandemic? Lessons for the Next Coronavirus Plague

**DOI:** 10.3390/biomedicines12010062

**Published:** 2023-12-27

**Authors:** Daniel López, Marina García-Peydró

**Affiliations:** 1 Presentation and Immune Regulation Unit, Centro Nacional de Microbiología, Instituto de Salud Carlos III, 28220 Majadahonda, Spain; 2 Centro de Biología Molecular Severo Ochoa, CSIC-UAM, 28049 Madrid, Spain; mgpeydro@cbm.csic.es

**Keywords:** HLA, vaccines, cross-reactivity, T cells, SARS-CoV-2

## Abstract

SARS-CoV-2 caused the devastating COVID-19 pandemic, which, to date, has resulted in more than 800 million confirmed cases and 7 million deaths worldwide. The rapid development and distribution (at least in high-income countries) of various vaccines prevented these overwhelming numbers of infections and deaths from being much higher. But would it have been possible to develop a prophylaxis against this pandemic more quickly? Since SARS-CoV-2 belongs to the subgenus sarbecovirus, with its highly homologous SARS-CoV-1, we propose here that while SARS-CoV-2-specific vaccines are being developed, phase II clinical trials of specific SARS-CoV-1 vaccines, which have been in the pipeline since the early 20th century, could have been conducted to test a highly probable cross-protection between SARS-CoV-1 and SARS-CoV-2.

## 1. Brief Introduction on SARS-CoV-2, COVID-19 and Vaccines

Coronavirus disease 2019 (COVID-19), caused by the SARS-CoV-2 viral pathogen, is a contagious disease responsible for an overwhelming pandemic, with more than 767 million confirmed infections and nearly 7 million deaths worldwide to date (https://www.who.int/emergencies/diseases/novel-coronavirus-2019/situation-reports, accessed on 1 November 2023). This virus is the third betacoronavirus, and the second within the sarbecovirus subgenus, to cause zoonoses in the last two decades.

After the World Health Organization (WHO) declared the COVID-19 pandemic a public health emergency in January 2020, the shock of observing collapsed hospitals and an exponential growth of contagions, sick people and deaths, in April 2020, almost half of the world’s population was under some kind of lockdown ordered by the different governments of more than 90 countries or territories (https://www.euronews.com/2020/04/02/coronavirus-in-europe-spain-s-death-toll-hits-10-000-after-record-950-new-deaths-in-24-hou, accessed on 1 November 2023). During that terrible spring of 2020, scientists from all over the world, different pharmaceutical companies and health agencies from various countries, as well as international, public and private entities, began (sometimes collaborating, sometimes competing) a race against time to obtain a vaccine to control this epidemic as quickly as possible. Finally, in August 2021, just 18 months after the onset of the COVID-19 pandemic, the “Pfizer-BioNTech COVID-19 Vaccine” was approved by the FDA. Subsequently, the WHO has authorized the emergency use of 11 additional COVID-19 vaccines (https://covid19.trackvaccines.org/agency/who/, accessed on 5 October 2023). Although these vaccines have some acute and transient side effects, usually allergic reactions or heart and cerebrovascular disorders [1,2,3], this has not prevented them from being used worldwide. The rapid development and distribution (at least in high-income countries) of these vaccines prevented the overwhelming number of infections and deaths from COVID-19 from being much higher [4,5,6]. As envelope glycoproteins are often considered ideal targets for vaccines against various viruses, all of these licensed COVID-19 vaccines contained the spike protein as the immunogen. However, despite this very rapid development, could another vaccine against COVID-19 have been developed even faster?

## 2. Empirical Vaccination against Smallpox Provides the First Clues

Among the many pandemics that have plagued mankind throughout history, few have had such a global and sustained impact on the human population as smallpox [7]. Thanks to its very striking and specific symptoms, efforts to control this pandemic by pre-scientific inoculation of smallpox scabs or pus were developed in ancient times in several Eastern cultures. And slowly, this empirical procedure spread over the centuries to virtually all historical societies [8]. However, as variolation used the live virus, people inoculated with scabs could transmit the disease to relatives and neighbors, initiating a new epidemic outbreak and a new cycle of deaths.

Throughout the 18th century, different Dutch, English and German physicians, and even some far-sighted farmers of these nations, correctly deduced that from the lack of vulnerability of milkmaids to smallpox, widely known in European rural areas dedicated to cattle farming, an effective and safe preventive treatment against this pandemic could be developed [8]. Finally, experiments conducted by Edward Jenner demonstrated person-to-person protection, and his perseverance in reporting his studies to the Royal Society initiated the era of prophylactic vaccines [9]. In this first empirical vaccination, two poxviruses (cowpox and horsepox) were used interchangeably. A century and a half later, the WHO-coordinated global mass vaccination program, adopted thanks to the tenacious insistence of the Soviet virologist Viktor Zhdanov, succeeded in eradicating this pandemic using the vaccinia virus, another poxvirus related to cowpox, horsepox and smallpox itself [10]. In addition, some studies suggest that vaccination with the vaccinia virus may even protect against recently detected new monkeypox infections [11,12].

## 3. Among Ebolaviruses, There Is Also Cross-Protection

The genus ebolavirus includes six virus species named for the region where each was originally identified: *Bombali ebolavirus*, *Bundibugyo ebolavirus*, *Reston ebolavirus*, *Sudan ebolavirus*, *Taï Forest ebolavirus* and *Zaire ebolavirus*. In a very recent article, Canadian and U.S. researchers tested the heterologous immunity hypothesis with two Ebolaviruses: *Sudan* and *Zaire*. In the guinea pig model, the authors demonstrated that 60% of animals vaccinated with a recombinant vesicular stomatitis virus (rVSV) engineered to express heterologous *Zaire ebolavirus* viral glycoprotein survived the challenge with *Sudan ebolavirus* [13]. These results indicated limited but relevant cross-protection in the rodent model. Previously, immunization of mice with rVSV expressing the glycoprotein of *Taï Forest* or *Reston* viruses generated complete cross-protection against mouse-adapted *Zaire ebolavirus* [14].

In summary, empirical vaccination against smallpox, as well as studies with viruses of the genus ebolavirus, indicate that vaccines against closely related viruses can induce complete or partial, but significant, cross-protective immunity.

## 4. Can There Be Cross-Protection between SARS-CoV-1 Vaccines and SARS-CoV-2 Infection?

Since the early 20th century, several SARS-CoV-1 vaccines, using inactivated SARS coronavirus (SARS-CoV-1 Sino 3 strain) or DNA constructions (VRC-SRSDNA015-00-VP), were in the pipeline. In preclinical studies, these vaccines were potent inducers of cellular and humoral immune responses and protective immunity [15,16,17]. Next, these vaccines caused no significant side effects in subjects and generated high neutralizing antibody titles and strong cellular immune responses in phase I clinical trials [18,19,20]. Further development of these clinical trials was halted with the end of the SARS-CoV-1 outbreak. However, as SARS-CoV-1 and SARS-CoV-2 are two highly homologous coronaviruses included in the sarbecovirus subgenus, would vaccines against SARS-CoV-1 be expected to protect against SARS-CoV-2 similar to the cases of smallpox and ebolaviruses mentioned above?

The staggering polymorphism of tens of thousands of human leukocyte antigen (HLA) class I and II alleles makes experimental testing of this hypothesis at the global population level extraordinarily difficult. However, these HLA alleles have been grouped into HLA class I and II families, superfamilies and supertypes, sharing strong similarities at the level of peptide ligand specificity. Thus, in our previous study, we performed a computational analysis of herd immunity that included the 600 most common HLA alleles, covering >90% of the world’s population, regardless of ethnicity. This in silico approach revealed that each of these frequent HLA alleles may be capable of presenting approximately 4-5 epitopes shared by SARS-CoV-1 vaccines and the SARS-CoV-2 spike protein [21]. In support of our findings, the limited data available from SARS-CoV-2 patients expressing a few of the most prevalent HLA alleles showed up to 91% concordance with the epitopes predicted in our study [21]. Since each individual expresses between 6 and 12 different alleles (6 per chromosome), a fully heterozygous individual, like 85% of the world’s population, could have up to 52 conserved viral epitopes between SARS-CoV-1 vaccines and the SARS-CoV-2 spike protein [21]. This high number of conserved epitopes between SARS-CoV-1 and SARS-CoV-2 is due to the fact that although the spike proteins of both coronaviruses show 304 changes, these are not randomly distributed but concentrated in specific regions of the viral protein. Thus, there are 27 segments containing between 9 and 111 consecutive conserved residues among SARS-CoV spike proteins. This allows the immune system to have access to a total of 579 amino acids from the SARS-CoV-1 spike protein to generate HLA-restricted epitopes that are conserved with SARS-CoV-2. In addition, a study in mice showed that immunization with SARS-CoV-1 vaccines induced not only cytotoxic and helper T lymphocytes specific for conserved SARS-CoV-2 epitopes, as we had hypothesized, but also some cross-reactive antibodies [22]. This partial cross-protective immune response of the entire adaptive immune system [22] also greatly strengthens our argument. In addition, SARS-CoV-2 vaccines also induced cross-reactive immune responses against SARS-CoV-1 and other coronaviruses in humans [22].

Importantly, the evolutionary pressure on SARS-CoV-2 since its transformation from a local pathogen to a global pandemic has generated a greater number of novel variants and subvariants. Therefore, can SARS-CoV-1 vaccines be expected to cross-protect against new SARS-CoV-2 variants? Because the new SARS-CoV-2 variants differ from the original Wuhan-1 strain by a few dozen changes, which are not randomly distributed but concentrated in a few regions of the spike sequence, our two previous immunoinformatics analyses showed that the vast majority of T cell epitopes remained conserved between the different variants and the Wuhan-1 strain-based vaccines: for example, 91% of the epitopes remained conserved for Omicron [23,24].

In summary, similar to smallpox and ebolaviruses, there would be sufficient cross-reactivity among sarbecoviruses to induce partial but significant cross-protective immunity.

## 5. SARS-CoV-1 Vaccines Could Have Been Rapidly Deployed as a First Line of Defense against the COVID-19 Pandemic

If a bioinformatics analysis like the one in our study [21] had been performed at the beginning of the COVID-19 pandemic, it would have been possible to predict cross-reactivity and subsequent partial protection between SARS-CoV-1 vaccines and SARS-CoV-2 infection. At the turn of the century, phase I trials of SARS-CoV-1 vaccines showed no significant side effects and were considered safe [18,19,20]. Thus, both data could then have allowed these vaccines to move into phase 2 and 3 clinical trials to test their potential use against COVID-19 in early 2020. Their expected partial efficacy would have made them suitable to reduce COVID-19 mortality from late 2020 until specific SARS-CoV-2 vaccines were licensed one year later. In this context, the malaria vaccine is an interesting example of a WHO-approved vaccine that, although only partially protective (30% of severe cases), is expected to prevent tens of thousands of deaths per year [25]. Therefore, given the explosive spread of SARS-CoV-2 during the pandemic, it is very likely that the number of lives saved during the year in which COVID-19-specific vaccines would have been in use would have been a relevant fraction of the millions of deaths that occurred during that time.

## 6. Fighting Future Pandemics with Bioinformatics Tools

For future pandemics, bioinformatics analyses as described above could be a useful, rapid and cost-effective strategy to determine the potential cross-reactivity of vaccines currently available or in development. And going even further, in the face of likely new and future unknown pandemics related to current zoonoses, could this strategy be used preventively?

In addition to the two zoonotic sarbecoviruses, MERS-CoV, another betacoronavirus included in the subgenus merbecovirus, also caused zoonotic disease in 2017. Due to the evolutionary divergence between the two subgenera, there are no conserved epitopes between MERS-CoV and sarbecoviruses for any HLA class I or II allele [21]. This means that if a new merbecovirus zoonosis were to emerge, as SARS-CoVs did, the currently licensed SARS-CoV-2 vaccines would likely not cross-protect against this new coronavirus. Since several close relatives of MERS-CoV identified in bats can effectively bind to angiotensin-converting enzyme 2 and use it as an entry receptor to infect human cells, the emergence of a new human pandemic merbecovirus is not inconceivable [26].

As a recombinant modified vaccinia virus, Ankara, currently in development as a specific vaccine against MERS-CoV, has demonstrated safety, tolerability and specific cellular and humoral responses in phase I clinical trials [27]. The WHO and other international organizations should consider the bioinformatics strategy in the face of a new zoonotic merbecovirus. Because with a mortality rate of 35%, like that of MERS-CoV, very rapid development of a vaccine against other merbecoviruses may be essential to avoid a dramatic scenario. Keep in mind that if a pandemic like COVID-19, with a mortality rate of about 5%, was capable of collapsing the health systems of developed countries and crippling the world economy, there may not be time to develop a specific vaccine against new zoonotic merbecoviruses from the outset. And then, the temporary use of the vaccine against MERS-CoV may provide sufficient partial cross-protection to allow time for the development of a specific vaccine against the new merbecovirus zoonosis.

## 7. Conclusions and Future Directions

Successful empirical vaccination against smallpox with related orthopoxviruses, cross-protection between different ebolaviruses and specific vaccines, and cross-reactive immune responses and partial cross-protection between SARS-CoV-1 and SARS-CoV-2 vaccines and sarbecoviruses have demonstrated that vaccines against one pathogen could be used against other closely related viruses. Thus, when facing new pandemics, bioinformatics analyses could be a useful, rapid and cost-effective strategy to determine the potential cross-reactivity of vaccines currently available or in development. This fact is particularly important in the case of pandemics with rapid spread and high mortality rates, where the fast availability of a vaccine (even if it provides only partial protection) may be critical to preserving public health systems and avoiding socioeconomic collapse.
